# Association of thyroid nodules with comorbidity burden and prognosis in patients with heart failure, anemia, and hyperuricemia: a retrospective cohort study

**DOI:** 10.3389/fendo.2026.1790376

**Published:** 2026-03-11

**Authors:** Da Huang, Jingwen Liang, Yi Zhou, Xingshou Pan, Zhengjiang Liu

**Affiliations:** Department of Cardiology, Affiliated Hospital of Youjiang Medical University for Nationalities, Youjiang Medical University for Nationalities, Baise, Guangxi, China

**Keywords:** anemia, anxiety, chronic heart failure, depression, serum uric acid, thyroid nodule

## Abstract

**Background:**

The prognostic role of thyroid nodules (TNs) in patients with heart failure with reduced ejection fraction (HFrEF) complicated by anemia and hyperuricemia (HUA) remains unclear. This study aimed to investigate the association of TNs with cardiovascular comorbidity burden and long-term prognosis in this high-risk population.

**Methods:**

A retrospective cohort of 185 inpatients with HFrEF, anemia, and HUA (2018-2022) was divided into TN (n=94) and non-TN (n=91) groups based on ultrasound findings. Baseline characteristics, laboratory parameters (thyroid function, coagulation), and psychological status (Hospital Anxiety and Depression Scale, HADS) were compared. The primary composite endpoint included all-cause mortality, heart failure rehospitalization, and cardiovascular events. Multivariable logistic and Cox regression analyses identified factors associated with TNs and prognosis.

**Results:**

The TN group had higher prevalence of coronary heart disease (65.9% vs 31.8%), atrial fibrillation (71.3%vs 27.5%), and depression scores (8.32 vs 6.02). Logistic regression identified depression (OR = 4.81, 95%CI 2.56-9.41), atrial fibrillation (OR = 4.46, 95%CI 2.09-9.51), and coronary heart disease (OR = 2.45, 95%CI 1.32-4.54) as independent factors associated with TNs. Depression and anxiety scores positively correlated with HbA1c (P<0.01) and negatively with NT-proBNP (P<0.05). During median follow-up of 21.8 months, 99.5% patients experienced adverse outcomes. Cox regression showed atrial fibrillation (HR = 1.95, 95%CI 1.35-2.80), diabetes (HR = 1.32, 95%CI 1.02-1.70), and prothrombin activity (HR = 1.01, 95%CI 1.00-1.01) as independent risk factors. Depression was associated with shorter median survival (14.0 vs 18.0 months).

**Conclusion:**

Thyroid nodules are associated with greater cardiovascular comorbidity burden, depression, and poorer prognosis in HFrEF patients with anemia and HUA. This association highlights the need for comprehensive management that includes cardiovascular risk assessment and psychological evaluation.

## 1 Introduction

Heart failure (HF) poses a significant global health challenge, affecting over 64 million people and ranking among the leading causes of morbidity and mortality worldwide. Despite advancements in pharmacological and device-based therapies, the prognosis for HF patients remains poor, with a first-year mortality rate ranging from 4% to 45% and an average mortality rate of approximately 33% (1–3). Anemia and HUA are common comorbidities in patients with heart failure and HFrEF, and are associated with more severe clinical symptoms and poorer outcomes in HF (4, 5).

Thyroid dysfunction is closely associated with cardiovascular system function, potentially affecting cardiac structure, function, and vascular endothelial function. For patients with pre-existing heart failure, thyroid function serves as a critical prognostic indicator (6). Chronic stress may induce abnormal proliferation and differentiation of thyroid cells, leading to nodule formation. As a structural alteration of the thyroid gland, thyroid nodules represent a clinically common thyroid disorder with increasing detection rates year by year (7, 8).

The relationship between these conditions and cardiovascular diseases has not been fully elucidated. Both anxiety and depression are recognized as issues in patients with HF, which adversely affect their prognosis. Early identification of cardiovascular risk factors associated with thyroid nodules and comorbid depression and anxiety is of significant importance for improving patient outcomes.

This study aims to investigate the correlation between emotional changes associated with thyroid nodules and the risk of HFrEF complicated by anemia and hyperuricemia, as well as patient prognosis, to provide theoretical basis for clinical diagnosis and treatment.

## 2 Methods

### 2.1 Study population

The study adopted a retrospective cohort design, collecting inpatients with HFrEF as the primary discharge diagnosis from the Department of Cardiovascular Medicine of our hospital from January 1, 2018, to September 1, 2022, totaling 185 cases of HFrEF patients with anemia and hyperuricemia. Inclusion criteria: (1) Meeting the European Society of Cardiology (ESC) diagnostic criteria for HFrEF (7); (2) Left ventricular ejection fraction (LVEF) <40% with elevated levels of N-terminal B-type natriuretic peptide precursor (NT-proBNP); (3) Age 33-85 years. Exclusion criteria: (1) Use of xanthine oxidase inhibitors, urate-lowering drugs, or colchicine; (2) History of thyroid treatment or thyroid dysfunction; (3) Severe hepatic or renal insufficiency; (4) Malignant tumors; (5) Active infectious diseases such as tuberculosis; (6) Severe mental disorders. This study was approved by our institutional review board and complies with the Declaration of Helsinki.

### 2.2 Definition of variables

The diagnosis of anemia was based on WHO standards: hemoglobin <120g/L in non-pregnant women and <130g/L in men. The diagnostic criteria for hyperuricemia referred to the “China Multidisciplinary Expert Consensus on the Diagnosis and Treatment of Hyperuricemia-Related Diseases” (2023 edition): fasting serum uric acid>420μmol/L on two separate occasions.

Ultrasonography: Two experienced ultrasound physicians independently analyzed thyroid nodule characteristics (size, orientation, margins, structure, echogenicity) and cardiac structural and functional parameters using color Doppler ultrasound diagnostic systems such as Philips EPIQ5 and Hitachi Ascendus. Discrepant results were arbitrated by a third physician.

Laboratory testing: Venous blood was collected from all study subjects 8 hours after fasting upon hospital admission. The complete blood count (CBC), thyroid hormone levels, and various biochemical parameters were measured using chemiluminescence immunoassay (CLIA). The testing equipment was an Abbott Automatic immune luminescence instrument(CLIA) from the United States, with reagents provided by Abbott Laboratories, Inc.

### 2.3 Anxiety and depression assessment

Within 48 hours after hospital admission, the anxiety and depressive symptoms of patients were assessed using the Chinese version of the Hospital Anxiety and Depression Scale. The scale consists of two subscales, each containing 7 items, for evaluating anxiety symptoms (HADS-A) and depressive symptoms (HADS-D), respectively. According to previous validation studies conducted in the China population, when the score of a subscale reaches or exceeds 8 points, it is considered to indicate the presence of clinically significant psychological problems (9).

Patients who met the diagnostic criteria for thyroid nodules were divided into the thyroid nodule group (94 cases) and the non-thyroid nodule group (91 cases) based on ultrasound diagnostic standards and laboratory tests.

Baseline data on demographics, clinical characteristics, laboratory tests, and pharmacotherapy were retrieved from hospital records. The primary composite endpoints included all-cause mortality, rehospitalization for heart failure, and cardiovascular events (acute coronary syndrome, stroke). Data were collected through electronic medical record reviews and telephone follow-ups, with follow-up continuing until January 31, 2024.

### 2.4 Statistical analysis

Statistical analysis was performed using SPSS (IBM SPSS, Inc., Armonk, NY, USA) and Prism (GraphPad Software, Inc., San Diego, CA, USA). Continuous measurement data were first tested for normality using the Shapiro–Wilk test. If normal distribution was met, data were expressed as mean ± standard deviation ( $\bar{\text{X}}$ ± S), and intergroup comparisons were conducted using the Student’s t-test. For comparing demographic characteristics between two groups, continuous data were analyzed using the Wilcoxon–Mann–Whitney test, while categorical data were presented as percentages and analyzed using theχ²test or Fisher’s exact test to assess differences. Spearman correlation analysis was employed to investigate the correlation between anxiety and depression in patients with thyroid nodules and their impact on heart failure with anemia and hyperuricemia (HUA). ROC curve analysis was used to evaluate the diagnostic value of anxiety and depression in the diagnosis of thyroid nodules. Kaplan-Meier survival analysis was employed to assess long-term outcomes in the two groups. To identify the emotional variables of anxiety and depression, univariate analysis of related variables was performed (*P ≤* 0.1) among patients with HFrEF, anemia, HUA, and thyroid nodules to determine cardiovascular risk factors. Significant variables were included in multivariate Cox regression analysis to identify independent predictors of adverse outcomes, with a bilateral *P* < 0.05 threshold for statistical significance. To ensure the stability of the multivariable models, multicollinearity among covariates was assessed using the Variance Inflation Factor (VIF), with a threshold of VIF < 5 indicating no significant collinearity. Furthermore, to address potential overfitting given the number of covariates relative to the sample size and event count, the Events Per Variable (EPV) ratio was calculated. We adhered to the principle of parsimony, selecting variables based on statistical significance (*P* < 0.1 in univariate analysis) and clinical relevance, ensuring an acceptable EPV ratio.

## 3 Results

### 3.1 Baseline clinical characteristics and comorbidities of HFrEF patients with anemia and hyperuricemia, stratified by thyroid nodule status

A total of 185 patients meeting the inclusion and exclusion criteria were enrolled in this study, including 94 cases (49.2%) in the thyroid nodule group and 91 cases (50.8%) in the non-thyroid nodule group. Compared to baseline data, patients in the thyroid nodule group exhibited higher prevalence of overweight, severe depression, and elevated depression incidence. Significant statistical differences were observed in the incidence rates of coronary heart disease (CHD), hypertension, atrial fibrillation (AF), and diabetes mellitus between the two groups (*P* < 0.05 or *P* < 0.01) (see **Table 1**).

**Table 1:** Baseline clinical characteristics and comorbidities of HFrEF patients with anemia and hyperuricemia, stratified by thyroid nodule status.

Variable	Thyroid nodule group (%)	NO thyroid group (%)	*t/Z/χ2*	*p*
Female	30 (31.9)	27 (29.7)	0.462	0.497
Male	64 (68.1)	64 (70.3)		
Age (yeas)	71.472 ± 7.75	70.29 ± 10.36	6.321	0.253
Weight (Kg)	59.48 ± 10.96	55.13 ± 8.09	9.123	0.000^***^
Pulse Rate (bpm)	85.56 ± 18.25	91.40 ± 20.24	0.038	0.008^**^
Systolic blood pressure (mmHg)	135.32 ± 27.16	129.55 ± 25.65	0.403	0.055
Dystolic blood pressure (mmHg)	80.84 ± 13.38	80.98 ± 17.33	4.829	0.939
Heart Rate (bpm)	90.42 ± 20.79	94.19 ± 23.39	0.579	0.133
Somker	47 (50)	44 (48.3)	0.180	0.671
Co-morbidities				
Coronary heart disease	62 (65.9)	29 (31.8)	13.581	0.000^***^
Hypertension	61 (64.5)	30 (32.9)	7.205	0.007^**^
Atrial fibrillation	67 (71.3)	25 (27.5)	3.994	0.046^*^
Valvular Heart Disease	36 (38.2)	55 (60.4)	2.686	0.101
Cardiomyopathy	48 (51.1)	43 (47.3)	1.205	0.272
Diabetes mellitus	41 (43.6)	50 (54.9)	6.302	0.012^*^
Renal insufficiency	57 (60.6)	34 (37.4)	1.346	0.246
NYHA class				
III	49 (52.1)	53 (58.2)	0.166	0.684
IV	42 (48.9)	41 (45.1)		
Depression	8.32 ± 0.43	6.02 ± 0.18	4.931	0.000^***^
Anxious	4.96 ± 0.31	4.46 ± 0.20	1.292	0.198
Aspirin	59 (62.8)	22 (24.2)	4.710	0.030^*^
Clopidogrel	63 (67.0)	28 (30.8)	7.348	0.007^**^

Data are presented as mean ± standard deviation (SD) for continuous variables with normal distribution, median (interquartile range) for non-normally distributed variables, and number (percentage) for categorical variables. Data are presented as mean ± standard deviation or number (percentage). Group comparisons were performed using Student’s t-test or the Mann-Whitney U test for continuous variables, and the Chi-square test or Fisher’s exact test for categorical variables, as appropriate. ***p < 0.001, **p < 0.01, *p < 0.05.

### 3.2 Comparison of admission laboratory parameters between HFrEF patients with and without thyroid nodules

Comparative analysis of baseline laboratory parameters (including routine and biochemical tests) between the two groups revealed statistically significant differences (*P* < 0.05) in the thyroid nodule group: Mean corpuscular volume (MCV), prothrombin activity, fibrinogen levels, and the ratio of free triiodothyronine (FT3) to free thyroxine (FT4) were higher than those in the non-nodule group. Conversely, total bilirubin, direct bilirubin, FT4, and aortic regurgitation area showed statistically significant differences (*P* < 0.05 or *P* < 0.01) in the nodule group. No significant differences were observed in other laboratory parameters (P>0.05) (see **Table 2**).

**Table 2:** Comparison of admission laboratory parameters between HFrEF patients with and without thyroid nodules.

Variable	Thyroid nodule group	NO thyroid group	t/Z	*p*
MCV(µm3)	89.49 ± 8.77	86.21 ± 12.35	16.65	0.038^*^
Prothrombin activity (%)	73.78 ± 23.59	67.01 ± 21.14	0.033.	0.042^*^
Fibrinogen(g/L)	3.78 ± 1.04	3.41 ± 1.09.	0.00.1	0.023^*^
Total bilirubin(µmol/L)	10.11 ± 0.82	13.02 ± 1.18	10.54	0.044^*^
Direct bilirubin (µmol/L)	5.01 ± 0.42	6.87 ± 0.67	12.37	0.020^*^
FT4(pmol/L)	17.13 ± 3.29	18.44 ± 3.15	0.971	0.006^**^
Aortic regurgitation area(cm^2^)	2.59 ± 0.31	3.95 ± 0.55	6.827	0.015^*^
FT3/FT4	0.22 ± 0.07	0.19 ± 0.06	1.813	0.002^**^

Data are presented as mean ± standard deviation (SD) or median (interquartile range) depending on data distribution. **p < 0.01, *p < 0.05.

### 3.3 Multivariable logistic regression analysis of clinical factors associated with the presence of thyroid nodules

In the multivariate stepwise logistic regression analysis (Forward: LR), with the presence of thyroid nodules (0=absent, 1=present) as the dependent variable, we aimed to identify independent clinical factors associated with this comorbidity, rather than to develop a diagnostic prediction model. After adjusting for confounding factors, seven variables emerged as statistically significant independent correlates. As shown in **Table 3**, depression (OR = 4.814, 95% CI: 2.561-9.408, *p* < 0.001), atrial fibrillation (OR = 4.457, 95% CI: 2.089-9.508, *p* < 0.001), coronary heart disease (OR = 2.447, 95% CI: 1.319-4.542, *p* = 0.005), and clopidogrel use (OR = 2.381, 95% CI: 1.272-4.458, *p* = 0.007) were identified as factors significantly associated with a higher likelihood of having thyroid nodules. Regarding continuous variables, higher levels of FT4 were positively associated with the presence of nodules (OR = 1.137, 95% CI: 1.038-1.246, *p* = 0.006), whereas higher body weight (OR = 0.964, 95% CI: 0.931-0.997, *p* = 0.034) and higher fibrinogen levels (OR = 0.621, 95% CI: 0.463-0.833, *p* = 0.001) were negatively associated. A higher pulse rate also showed a significant but weaker positive correlation (OR = 1.019, 95% CI: 1.002-1.037, *p* = 0.028) (see **Table 3**). Collinearity diagnostics for the logistic regression model revealed no severe multicollinearity among the included predictors, as all Variance Inflation Factors (VIFs) were well below the cut-off value of 5 (ranging from 1.02 to 1.45). This confirms that “Depression” did not exhibit significant collinearity with other clinical parameters. Additionally, the model’s reliability was supported by an adequate number of outcome events (94 thyroid nodule cases) relative to the 7 covariates included, yielding an Events Per Variable (EPV) ratio of approximately 13.4, which exceeds the recommended minimum of 10 to minimize overfitting risks.

**Table 3:** Multivariable logistic regression analysis of clinical factors associated with the presence of thyroid nodules.

Variable	B	SE	Wald	*df*	*p*	Exp(B)	95% EXP (B) CI
Pulse Rate (bpm)	0.019	0.009	4.802	1	0.028	1.019	1.002-1.037
Weight(Kg)	-0.037	0.017	4.507	1	0.034	0.964	0.931-0.997
Fibrinogen level (g/L)	-0.477	0.150	10.128	1	0.001	0.621	0.463-0.833
FT4 (pmmol/L)	0.129	0.047	7.546	1	0.006	1.137	1.038-1.246
Atrial fibrillation	1.494	0.387	14.942	1	<0.001	4.457	2.089-9.508
Coronary heart disease	0.895	0.316	8.023	1	0.005	2.447	2.089-9.508
Depression	1.571	0.322	23.817	1	<0.001	4.814	2.561-9.408
Clopidogrel	0.868	0.320	7.356	1	0.007	2.381	1.272-4.458

Multivariable logistic regression analysis was performed to identify factors associated with the presence of thyroid nodules (Dependent variable: 0 = Absent, 1 = Present). Variables with *P* < 0.05 in this multivariable model were considered independent factors associated with thyroid nodules, P < 0.05 indicates statistical significance.

### 3.4 Correlation analysis of metabolic and cardiac function indexes with mood disorders in two groups of patients

**Figure 1:**
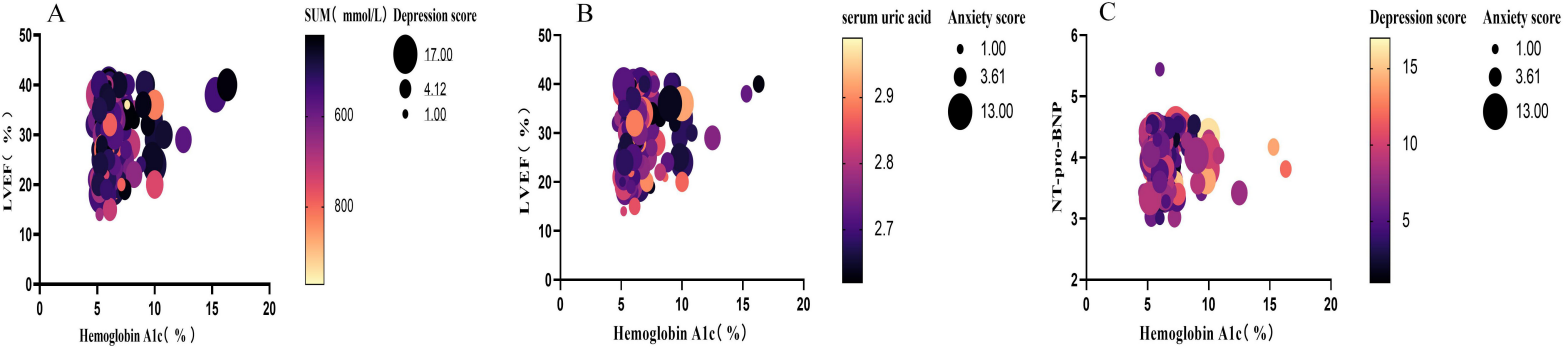
Correlation analysis between mood disorders, metabolic, and cardiac function indices. **(A)** Scatter plot showing the significant positive correlation between depression and anxiety scores (r = 0.441, P < 0.001). **(B, C)** Scatter plots demonstrating the correlations of depression and anxiety scores with HbA1c (positive correlation) and NT-proBNP (negative correlation), respectively. Uric acid showed a negative correlation with LVEF and a positive correlation with NT-proBNP, but no significant association with mood scores.

Pearson correlation analysis was performed to evaluate the associations between glycemic metabolism, uric acid, cardiac function indices, and mood disorders. The results showed (see **Figures 1A–C**) that depression and anxiety were highly positively correlated (r = 0.441, *P* = 0.000). Depression and anxiety scores were positively correlated with glycated hemoglobin (r = 0.165, *P* = 0.003; r = 0.145, *P* = 0.008) and negatively correlated with Nt-pro-BNP (r = -0.139, *P* = 0.012; r = -0.146, *P* = 0.008). Uric acid was negatively correlated with LVEF (r = -0.175, *P* = 0.001) and moderately positively correlated with NT-proBNP (r = 0.280, *P* < 0.001); it showed no correlation with anxiety or depression. Elevated uric acid levels were associated with cardiac dysfunction and increased NT-proBNP, while chronic hyperglycemia (HbA1c) was positively correlated with mood disorders. Abnormal mood was negatively associated with cardiac neurohormone activation (NT-proBNP), suggesting the coexistence of a “metabolism-cardiac function-mood” triad in this cohort (see **Figures 1A–C**).

### 3.5 Depression score demonstrates predictive value for thyroid nodule presence

**Figure 2:**
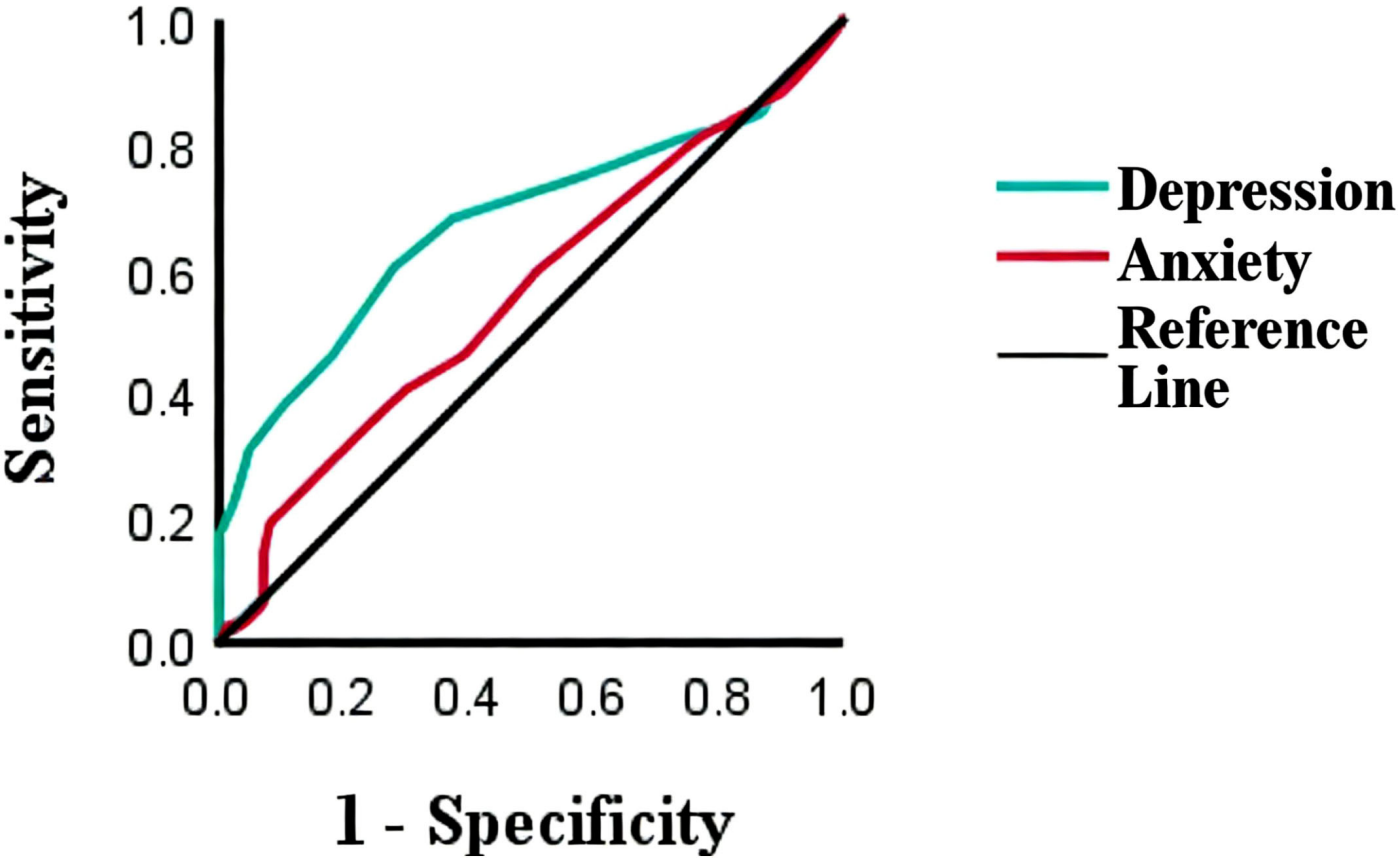
Receiver operating characteristic (ROC) curve analysis for depression and anxiety scores in predicting thyroid nodules. The area under the curve (AUC) for depression was 0.676 (95% CI: 0.615-0.736), with a sensitivity of 84.6% and specificity of 86.5% at the optimal cutoff of 3.5 (*P* < 0.001). The AUC for anxiety was 0.559 (95% CI: 0.496-0.621, *P* = 0.066).

ROC curve analysis results showed that the area under the ROC curve for patients with depression was 0.676 (95% CI = 0.615–0.736), with a sensitivity of 84.6%, specificity of 86.5%, and the optimal cutoff value of 3.5 (*P* = 0.000). For patients with thyroid nodule anxiety, the area under the ROC curve was 0.559 (95% CI = 0.496–0.621), with a sensitivity of 88.5%, specificity of 90.6%, and the optimal cutoff value of 1.5 (*P* = 0.066). Neither group achieved extremely high diagnostic accuracy, but the depression scores in the depression group demonstrated good diagnostic value in predicting the formation of thyroid nodules (see **Figure 2**).

### 3.6 Depression is associated with significantly shorter survival in the study cohort in Cox regression analysis of survival time

During the median follow-up period of 21.78 months (interquartile range 19.42,24.14), a total of 181 patients (99.5%) experienced at least one confirmed adverse outcome, including 118 patients (63.8%) hospitalized due to progressive heart failure, 24 patients (13%) who died, and 38 patients (20.5%) who experienced cardiovascular events.

**Figure 3:**
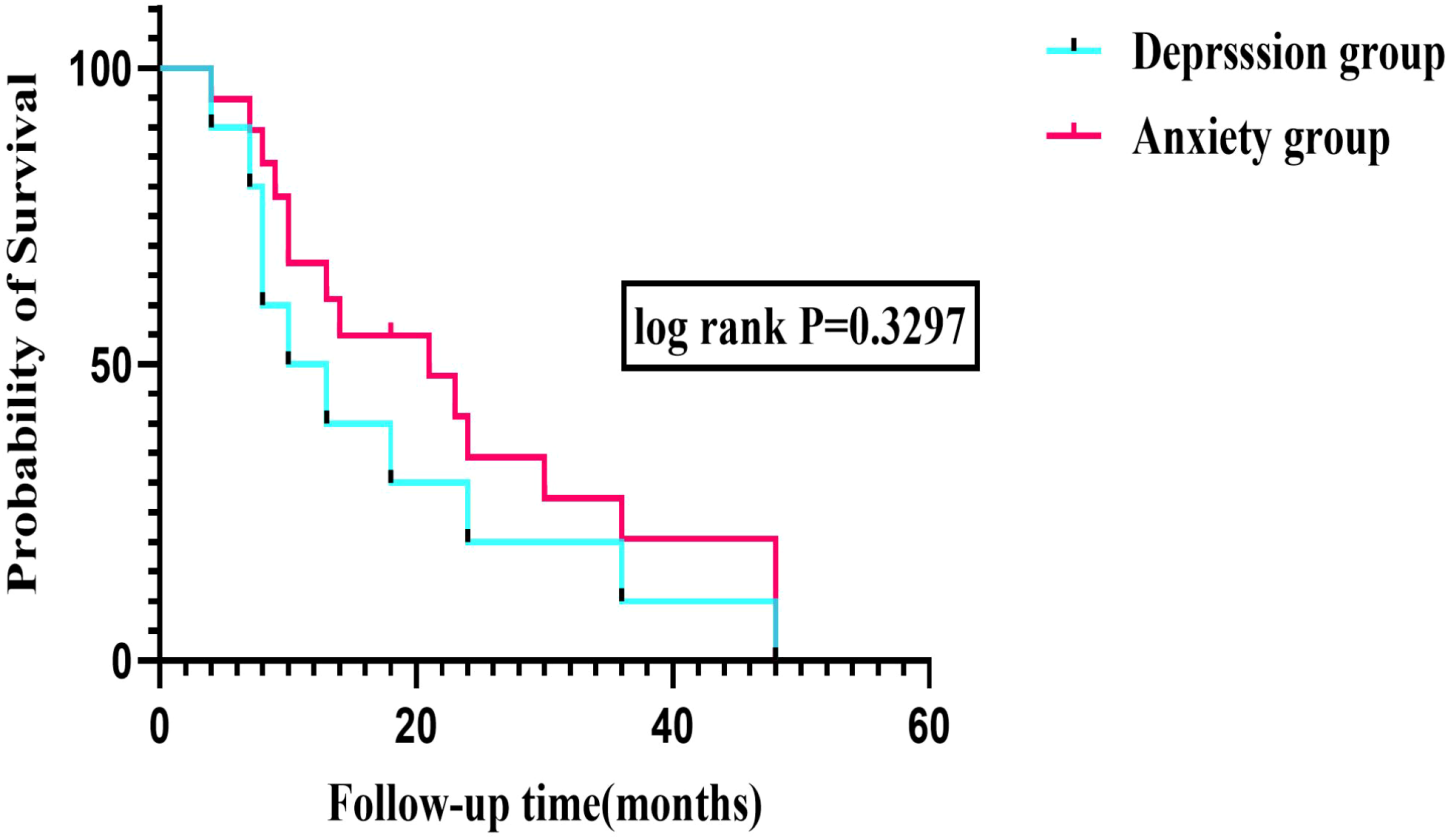
Kaplan-Meier survival curves comparing time to the composite endpoint between patients with and without depression. The median survival time was 14.0 months (95% CI: 9.8-18.2) in the depression group versus 18.0 months (95% CI: 14.7-21.3) in the non-depression group(Log-rank *P*-value =0.3297).

In Kaplan-Meier survival curve analysis, the median survival time was 14.0 months (95% CI: 9.8-18.2) in the depression group, 18.0 months (95% CI: 14.7-21.3) in the non-depression group, and 14.0 months in the anxiety group. The non-anxiety group showed the best median survival time of 20.0 months, which was the ‘gold standard’ group for prognosis. Depression was strongly associated with thyroid nodule risk and significantly shortened the ‘nodule-free survival period’ (**Figure 3**).

### 3.7 Atrial fibrillation, diabetes, and prothrombin activity independently predict adverse outcomes in multivariate Cox regression analysis

The independent prognostic value of comorbidities was assessed using a stepwise Cox proportional hazards model. Based on the results of univariate analysis, variables with significant differences were included in the Cox regression model. In cardiovascular comorbidities, the following variables were significantly associated with the risk of the end-point event (*P* < 0.05): prothrombin activity (HR = 1.007, 95% CI: 1.001–1.013, *P* = 0.017); history of diabetes (HR = 1.318,95% CI: 1.022–1.699, *P* = 0.033); history of atrial fibrillation (HR = 1.947,95% CI: 1.354–2.799, *P* < 0.001); fibrinogen level (HR = 1.115,95% CI: 0.097–1.248, *P* = 0.057); and free thyroid (HR = 1.037,95% CI: 0.998–1.077, *P* = 0.060) (See **Table 4**). -Similarly, in the Cox proportional hazards model, variance inflation factor analysis indicated the absence of multicollinearity (all VIFs < 3). The multivariate model included 6 covariates with 181 composite endpoint events observed during follow-up. This resulted in a robust EPV ratio (>20), suggesting that the model is unlikely to suffer from overfitting and that the identified independent prognostic factors are statistically reliable.

**Table 4:** Multivariable Cox proportional hazards regression analysis for predictors of the composite endpoint (all-cause mortality, HF rehospitalization, and cardiovascular events).

Variable	B	SE	Wald	*Df*	*p*	Exp(B)	95% EXP (B) CI
Prothrombin activity (%)	0.007	0.003	5.691	1	0.017	1.007	1.001-1.013
Fibrinogen (g/L)	0.109	0.057	3.618	1	0.57	1.115	0.097-1.248
FT4 (pmmol/L)	0.037	0.019	3.541	1	0.06	1.037	0.998-1.077
Diabetes mellitus	0.276	0.130	4.529	1	0.033	1.318	1.022-1.699
Atrial fibrillation	0.666	0.185	12.949	1	0.000	1.947	1.354-2.799

Multivariable Cox proportional hazards regression analysis was performed to identify independent predictors of the composite endpoint (all-cause mortality, heart failure rehospitalization, and cardiovascular events). The model was adjusted for age, sex, presence of thyroid nodules, history of coronary heart disease, and relevant comorbidities. The proportional hazards assumption was verified and satisfied. *P* < 0.05 was considered statistically significant.

## 4 Discussion

Thyroid nodules are the most common thyroid disorders. The results of this study indicate that patients with thyroid nodules tend to be overweight, with a high incidence of coronary heart disease (CHD) and hypertension, suggesting a significantly increased risk of cardiovascular diseases in these patients. This may be associated with potential subclinical thyroid dysfunction, inflammatory responses, and metabolic disturbances in thyroid nodules. These factors can promote the development and progression of cardiovascular diseases by affecting food intake, blood, lipid, and glucose metabolism, as well as vascular endothelial function, thereby influencing the prognosis of cardiovascular disease patients (10–18).

Anemia is a common manifestation of congestive heart failure (CHF). It is caused by cytokine-mediated bone marrow suppression and is associated with poor survival rates (19). Hyperuricemia is an independent predictor of poor prognosis in patients with acute or chronic HF, and it is independent of baseline left ventricular ejection fraction (LVEF) (20).

In this study of patients with HFrEF complicated by anemia and hyperuricemia, the progressive deterioration of left and right ventricular and atrial functions leads to thyroid hormone metabolic disturbances, affecting the synthesis of fibrinogen and prothrombin activity. This reflects the systemic fluid and metabolic deterioration in advanced heart failure, characterized by tachycardia, fluid retention, hepatic congestion, cholestasis, elevated bilirubin, reduced renal blood flow, malnutrition, and peripheral metabolic changes, all of which influence prognosis. These findings reveal the common metabolic and inflammatory pathophysiological basis underlying thyroid nodules (21). Cardiovascular diseases such as coronary artery disease (CAD), myocardial infarction (MI), heart failure, and hypertension may increase the risk of depression but do not elevate the risk of anxiety (18). Cardiovascular metabolic risk factors, including hyperuricemia and diabetes, also play a mediating role in the relationship between anxiety, depression, and cardiovascular diseases (22, 23).

There is a strong correlation between thyroid diseases, anxiety, and depression. The presence of thyroid nodules may itself have a negative impact on patients’ mental health, with individuals who have thyroid nodules being more prone to experiencing anxiety and depressive symptoms compared to those without nodules (24–26). The results of this study indicate that in patients with HFrEF complicated by anemia and hyperuricemia, depression emerged as the factor most strongly associated with the presence of thyroid nodules. While the cross-sectional nature of our analysis precludes causal conclusions, this strong association suggests a potential shared pathophysiological pathway involving neuro-endocrine dysregulation, with an association strength surpassing that of traditional risk factors such as atrial fibrillation and coronary heart disease. The changes in depressive and anxious emotions in HFrEF patients with comorbidities exacerbate the pathological progression of heart failure through neuroendocrine and inflammatory pathways, ultimately affecting patient survival rates (27).

Within the normal thyroid function range, higher concentrations of FT4 are associated with an increased risk of atrial fibrillation and heart failure, while lower concentrations of FT4 are linked to a reduced risk of multiple adverse events, including mortality, in elderly individuals (12, 28–34). Notably, the results of this study indicate that in patients with HFrEF and thyroid nodules who also have anemia and hyperuricemia, coagulation function parameters, atrial fibrillation, and diabetes are independent risk factors for poor prognosis in this population. Changes in hemoglobin concentration during follow-up were not associated with prognosis.

Patients with heart failure with reduced ejection fraction (HFrEF) complicated by anemia and hyperuricemia exhibit mutually reinforcing disease interactions that significantly worsen prognosis and reduce survival rates. The potential subclinical thyroid dysfunction in these heart failure comorbidities involves distinct pathophysiological mechanisms, where thyroid nodules may influence patient outcomes through multiple pathways including depressive mood alterations. Whether the complex interplay between thyroid hormones and cardiac function exerts a bidirectional effect requires further investigation. In patients with complex comorbidities of HF, psychological factors must be analyzed, necessitating the most proactive and comprehensive comprehensive management in clinical practice.

## 5 Conclusions

In summary, thyroid nodules are associated with a heavier burden of cardiovascular comorbidities and poorer prognosis in patients with HFrEF complicated by anemia and hyperuricemia. Clinical attention should be paid to cardiovascular risk assessment in such patients, Cardiologists should pay extra attention to the thyroid when encountering patients with severe depression and atrial fibrillation, and comprehensive management strategies should be optimized to improve prognosis.

This study still has some limitations: First, thyroid nodules and thyroid function were only examined at admission, without thyroid autoantibody testing, and were not evaluated during follow-up. Second, this study was a single-center, retrospective, observational study with a relatively small sample size, which may introduce selection bias. Additionally, as a cross-sectional and retrospective study, it is difficult to establish a clear causal relationship. The study analyzes data from 2018 to 2022. As we are currently in 2026, there is a four-year gap that may slightly reduce the clinical or practical impact of the findings. Third, as a retrospective observational study, our findings establish statistical associations but cannot prove causality. For example, while we found a strong link between depression and thyroid nodules, we cannot determine whether depression contributes to nodule formation or if both are downstream manifestations of systemic stress in heart failure. Finally, some potential influencing factors were not included in the analysis, which may have affected the results. Given that the study period covers 2020-2021, we should consider whether the COVID-19 pandemic introduced data biases or anomalies. It would be beneficial to mention this as a specific limitation if deemed appropriate.

Future research should conduct multicenter, large-sample, prospective studies to further explore the relationship between thyroid nodules and cardiovascular diseases.

## Data Availability

The original contributions presented in the study are included in the article/supplementary material. Further inquiries can be directed to the corresponding author.
